# Human Chromosome Y and Haplogroups; introducing YDHS Database

**DOI:** 10.1186/s40169-015-0060-7

**Published:** 2015-06-10

**Authors:** Timo Tiirikka, Jukka S Moilanen

**Affiliations:** Department of Clinical Genetics, Oulu University Hospital, PEDEGO Research Unit, University of Oulu, and Medical Research Center Oulu, Oulu University Hospital and University of Oulu, PO Box 23, FI-90029 Oulu, Finland

**Keywords:** Y chromosome, Y-DNA, Database, SNP, Mutation, Haplogroup

## Abstract

**Background:**

As the high throughput sequencing efforts generate more biological information, scientists from different disciplines are interpreting the polymorphisms that make us unique. In addition, there is an increasing trend in general public to research their own genealogy, find distant relatives and to know more about their biological background. Commercial vendors are providing analyses of mitochondrial and Y-chromosomal markers for such purposes. Clearly, an easy-to-use free interface to the existing data on the identified variants would be in the interest of general public and professionals less familiar with the field. Here we introduce a novel metadatabase YDHS that aims to provide such an interface for Y-chromosomal DNA (Y-DNA) haplogroups and sequence variants.

**Methods:**

The database uses ISOGG Y-DNA tree as the source of mutations and haplogroups and by using genomic positions of the mutations the database links them to genes and other biological entities. YDHS contains analysis tools for deeper Y-SNP analysis.

**Results:**

YDHS addresses the shortage of Y-DNA related databases. We have tested our database using a set of different cases from literature ranging from infertility to autism. The database is at http://www.semanticgen.net/ydhs

**Conclusions:**

Y-chromosomal DNA (Y-DNA) haplogroups and sequence variants have not been in the scientific limelight, excluding certain specialized fields like forensics, mainly because there is not much freely available information or it is scattered in different sources. However, as we have demonstrated Y-SNPs do play a role in various cases on the haplogroup level and it is possible to create a free Y-DNA dedicated bioinformatics resource.

## Background

**Fig. 1:**
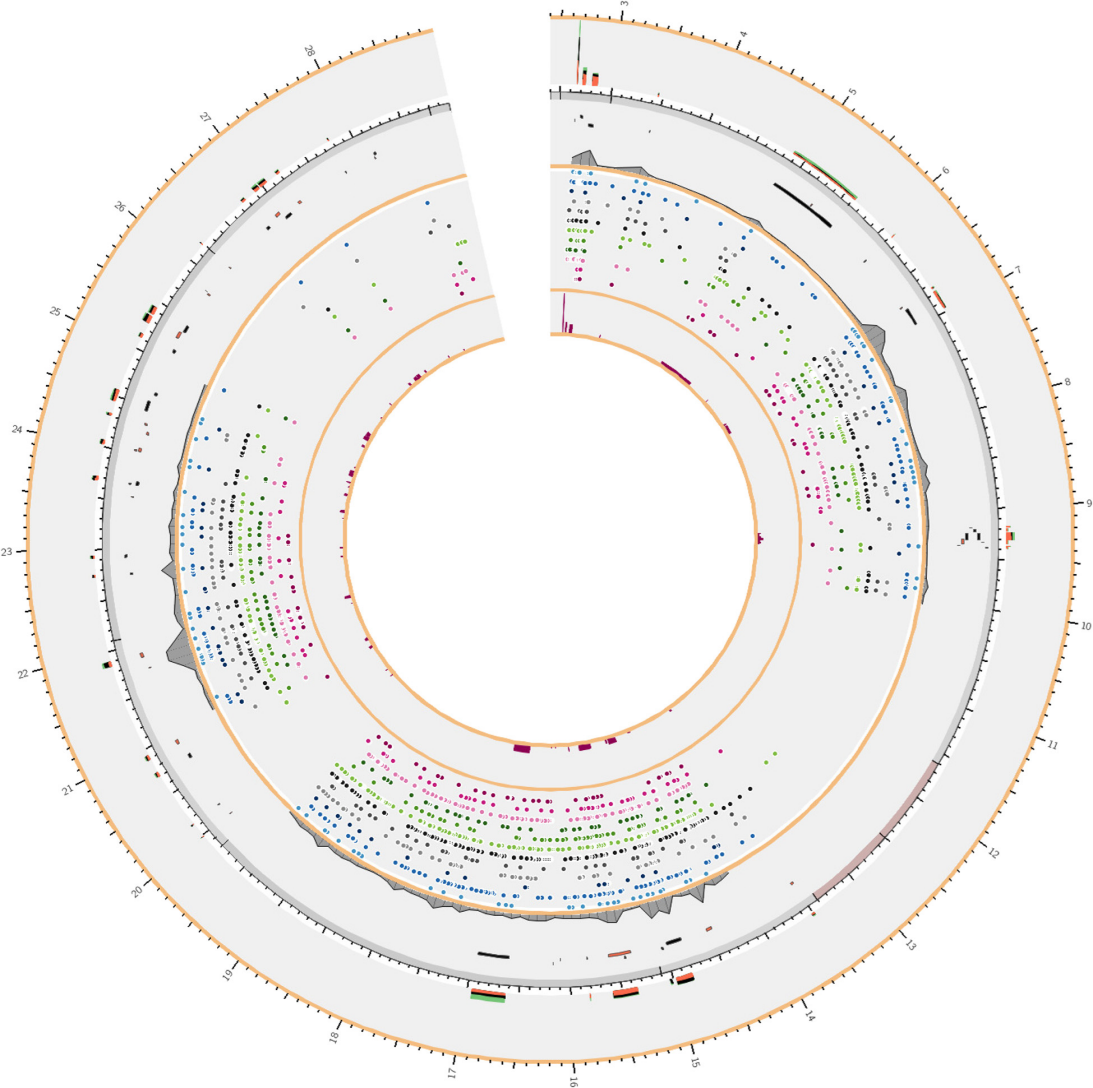
The image contains multiple tracks from YDHS database: starting from the outer rim with Gene Ontology (GO), Gene, SNP and finally the innermost track is OrphaNet. In the Gene Ontology track biological process is viewed in red, molecular function in black, and cellular component in green. The height of the GO bar corresponds to the amount of ontologies linked to the gene. In the Gene track the positive strand is colored black and the negative one in red. The third track counting from the outer rim is the SNP track where different types of SNPs have different color schema. From up to down: T- > G is in light blue, T- > C is in blue, T- > A is in dark blue; G- > T is in light grey, G- > is in grey, G- > A is in black; C- > T is in light green, C- > G is in green, C- > A is in dark green; A- > G is in pink, A- > C is in red, A- > T is in dark red. In the innermost track, OrphaNet and OMIM, the higher the bar is the more diseases has been reported for that gene or region. The grey plot on top of the SNP track indicates the frequency of SNPs in that region. There are some clearly visible 'hotspots' where a lot of mutations have clustered

The human Y chromosome has unique characteristics which make it the subject of intense research. Indeed, it has been 10 years now since the Y chromosome was initially sequenced in 2003. Unlike the 22 autosomal pairs of human chromosomes, the Y chromosome does not exist as a homologous pair. Therefore, the majority of the Y chromosome does not undergo recombination and is transmitted to male offspring in a virtually unchanged form, resulting in vertical transmission of haplotypes. Some genetic polymorphisms of the Y chromosome are associated with observed Y-linked phenotypic differences. The uniparental inheritance of mutations results in different “at risk” haplotypes among different paternal lineages [1], which are frequently dependent on ethnicity and the extent of admixture among populations. The field of medical genetics has utilized this pattern of heritability to study the relationship of specific mutations in Y chromosome genes and haplotypes which influence susceptibility to a number of of physical and mental disorders. The human Y chromosome consists of 86 genes coding 23 proteins (506 genes if pseudogenes are included) according to Ensembl e72 build. The genes in the Y chromosome are organized to specific chromosomal regions as illustrated in Fig. 1.

## Methods

**Fig. 2:**
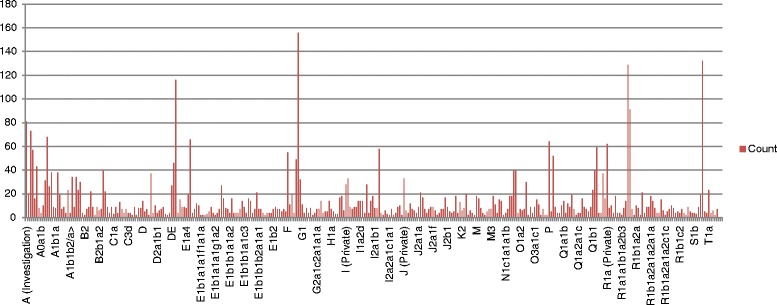
The frequency of mutations that hit gene regions separated by haplogroups. Data derived from ISOGG consortium (July 2013)

**Fig. 3:**
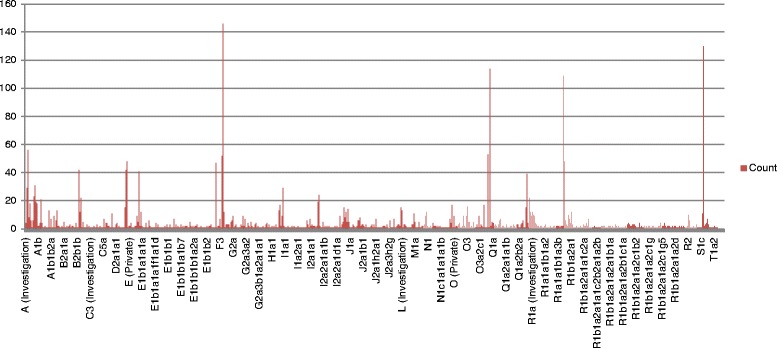
The overall distribution of mutations per haplogroup. Data derived from ISOGG on July 2013

We have developed a novel database YDHS (http://semanticgen.net/ydhs) to integrate haplogroup, genetic and genomic information regarding human chromosome Y. The database contains mutation and haplogroup data from the International Society of Genetic Genealogy (ISOGG; http://www.isogg.org/) consortium compiled in June 2013 (Fig. 1). The specific coverage of mutations per genes in haplogroups and the general distribution of mutations per haplogroup are illustrated in Fig. 2 and Fig. 3, respectively. We have developed an automated parser that transforms the Y-DNA haplotree and corresponding mutations with their positions in Grc37 build version to a MySQL database format for easy querying. Citations of the articles where SNPs were first introduced are also included in the database, and the citations are searchable via the web user interface, and a direct link is included if provided by ISOGG. Genomic information from Ensembl (GRch37) was integrated to our database using SNP positions to find out which SNPs are located inside genes and which involve amino acid sequences of the proteins. The genomic location can be visualized by clicking the mutation position, which opens up the location in the UCSC Genome Browser (http://genome.ucsc.edu/), or if there is a reference SNP available one can use the provided link to dbSNP (http://www.ncbi.nlm.nih.gov/SNP/). Gene Ontology (http://www.geneontology.org/), InterPro (http://www.ebi.ac.uk/interpro/) and UniProt (http://www.uniprot.org) data were added to the database using gene identifiers as unique keys, allowing the user to browse the functional information of genes and proteins by family based classification, domain and function prediction alongside the manually annotated entries to see what the gene/protein does in the human body. Relevant data about the potential diseases associated with the gene of interest were also added to the database. Hyperlinks to entries in the OMIM database (http://www.ncbi.nlm.nih.gov/omim) and OrphaNet (http://www.orpha.net) were associated with the respective genes. Egenetics (http://www.sanbi.ac.za/resources/software-downloads/evoc/) was used as the source of expression data separated by cell type, anatomical system and pathology. If the protein had a structure stored in Protein Data Bank PDB (http://www.rcsb.org) it was also hyperlinked in YDHS under the “Extra” tab.

The majority of the polymorphisms are located outside of coding regions of genes, and not all coding-region variants are pathogenic. The functional consequences of polymorphisms were assessed by using Variant Effect Predictor (VEP). Every polymorphism derived from the ISOGG database was subjected to VEP by Representitational State Transfer (REST) interface (http://rest.ensembl.org/) provided by EBI, the European Bioinformatics Institute. VEP consists of two predictor programs, SIFT (http://sift.jcvi.org/) and PolyPhen (http://genetics.bwh.harvard.edu/pph2/). VEP is good for predicting mutations affecting protein function, but additional tools to increase the prediction accuracy for rare polymorphisms were considered important. PROVEAN (http://provean.jcvi.org/) and Variation Reporter (http://www.ncbi.nlm.nih.gov/variation/tools/reporter) from NCBIs ClinVar toolkit were added to increase the prediction capabilities of YDHS. These tools used in sync can provide valuable information when the deleterious potential of a mutation is otherwise difficult to assess.

We also provide information whether the gene of interest is under positive selection using the Selectome database (http://selectome.unil.ch/). In addition, we provide information regarding repeats for the gene of interest via UCSC genome tracks. The location and population of the highest frequency of the haplogroup of interest is browseable by clicking the haplogroup (where) link provided that the information is available. The haplogroup location and population lists were compiled from Wikipedia (https://en.wikipedia.org/wiki/Human_Y-chromosome_DNA_haplogroup) on June 2013.

The proteomics part of YDHS consist of protein-protein interaction information via PSICQUIC (Proteomics Standard Initiative Common QUery InterfaCe) service as proposed by the HUPO Proteomics Standard Initiative. We have used EBIs PSICQUIC View to give us access to 28 different proteomics databases (http://www.ebi.ac.uk/Tools/webservices/psicquic/view/main.xhtml). The user can also visualize the protein-protein interaction graphs from STRING database in YDHS with one click from the menu. If the user would like to view the orthologs of the gene of interest, it can be done via the Gene IDs tab in the database. Ortholog entries come from InParanoid7 database (http://inparanoid.sbc.su.se).

The YDHS database is built on top of MySQL database and outputs the information as JavaScript Object Notation (JSON) which is parsed via HTML5 if the user’s browser is capable of handling it; otherwise standard AJAX calls are used. Ease of use was considered to be of importance and thus the user interface is simple and stripped from extraneous functionality commonly found on various core databases for biological information. YDHS provides a data export option in JSON format for computational parsing and for input to external analysis programs. The integration of different databases is done by dedicated PHP and Perl scripts. YDHS was used for the exploratory analysis of haplogroups and traits covered in this article. The Fig. 1 was drawn using Circos software (http://circos.ca/) and YDHS data.

## Results and discussion

Since there are altogether 3896 SNPs in the ISOGG Y-DNA tree (December 2013) and many more unsubstantiated ones, we decided to show the use of YDHS database using case examples. We chose four traits that have a linkage to Y chromosome according to the literature. Using these cases we tested how well YDHS can concur with already published results and whether the use of our integrative approach could yield additional information about the traits.

### Case: infertility

One of the most frequent subjects of Y chromosome-related medical genetics is male infertility. Microdeletions in the q arm are one of the most common causes of infertility, especially those which result in the loss of some or all of the azoospermia factor (AZF) gene [2, 3]. These mutations can result in oligospermia or azoospermia, cryptorchidism [4], and aneuploidy [5]. Although mutations on the Y chromosome should be preserved in male offspring that these men do produce, it is unclear whether specific haplotypes are associated with such microdeletions. Idiopathic reduced sperm counts are associated with microdeletions in Danish men with haplotype hg26 [6]. Hg26 is a rare haplotype among European males [7] and it may continue to decline if it remains associated with subfertility. 3 of 4 Japanese fertility patients with AZFa microdeletion were from the same haplogroup, D2b [8]. D2b is also associated with AZFc microdeletions [9]. However, this haplogroup is common in Japan [10] and the prevalence of microdeletions might be proportionate with prevalence of that haplotype.

The haplogroup D2b was refactored from ISOGG nomenclature in 2011, but the data is available as haplogroup D2a. Our YDHS database contains only one mutation for haplogroup D2a; namely M116.1. However, interestingly M116.1 is located inside TXLNG2P pseudogene. A recent study by Sato et al. [11] specified the haplogroup involded in azoospermia in Japanese men to be D2*. For that haplogroup there are 11 mutations in the database of which 8 are located inside gene regions and one in the TXLNG2P pseudogene. Chromosome Y hosts 23 AZF genes (Table 1). The AZF gene KDM5D has the greatest coverage of ISOGG mutations, namely 861, followed by DDX3Y with 320 mutations. In many cases, there exists no relationship between microdeletions, infertility, and haplotype [6, 12]. However, the use of YDHS facilitates linking mutations and genes in a haplogroup thus making it possible to compare subhaplogroups inside complex diseases like infertility.

**Table 1:** Shows the names of genes located in chromosome Y with the amount of respective mutations alongside with the frequency of GO terms

Ensembl ID	Gene name HGNC	No. ISOGG mutations	Positive selection?	No. Gene Ontologies	Gene length (bp)	MIM Gene Accession(s)	Orphanet Ids
ENSG00000012817	KDM5D	861	No	55	41,074	426,000	1646
ENSG00000067048	DDX3Y	320	No	21	16,371	400,010	-
ENSG00000114374	USP9Y	250	No	12	159,604	400,005	1646
ENSG00000129873	CDY2B	-	No	8	2810	400,018	-
ENSG00000131002	Cyorf15A / Cyorf15B	-	No	0	38,961	400,031	-
ENSG00000157828	RPS4Y2	12	No	6	24,868	400,030	-
ENSG00000169789	PRY	-	No	0	24,240	400,019,400,041	-
ENSG00000169807	PRY2	-	No	0	24,251	400,019,400,041	-
ENSG00000169953	HSFY2	-	No	18	42,295	400,029	-
ENSG00000172288	CDY1	-	No	10	2785	400,016	-
ENSG00000172468	HSFY1	-	No	24	42,292	400,029	-
ENSG00000182415	CDY2A	-	No	8	2810	400,016,400,018	-
ENSG00000183753	BPY2	-	No	10	31,646	400,013	-
ENSG00000183795	BPY2B	-	No	5	31,647	400,013	-
ENSG00000185894	BPY2C	-	No	5	31,647	400,013	-
ENSG00000187191	DAZ3	-	No	16	50,410	400,027	1646
ENSG00000188120	DAZ1	-	No	23	69,739	400,003	1646
ENSG00000198692	EIF1AY	108	No	7	17,429	400,014	-
ENSG00000205916	DAZ4	-	No	27	73,175	400,003,400,026	1646
ENSG00000205944	DAZ2	-	No	48	71,909	400,026	1646
ENSG00000234414	RBMY1A1	1	No	19	37,954	400,006	1646
ENSG00000244646	XKRY2	-	No	0	8417	-	-
ENSG00000250868	XKRY	-	No	0	8418	-	-

If the gene has a very high number of GO terms associated, it can be argued that the gene is functionally active and probably part of a signaling network. It can be stated that genes having a lot of mutations also have a very high number of ontologies, except for EIF1AY. Table 1 reveals also the length of the gene so that the density of mutations per gene can be addressed. The MIM and OrphaNet codes are also included to show how the genes are linked to various medical conditions

Other mechanisms of Y chromosome-associated male infertility have also been identified or hypothesized for subfertile but karyotypically normal males. Genomic instability is one such mechanism, in which either chromosomal or microsatellite instability can result in spermatic aneuploidy [13, 14]. A non-exclusive possibility is that dysfunctional androgen receptors may result in infertility in men with normal androgen levels [15]. These and other abnormalities which inhibit fertility could be inherited via nontraditional mechanisms (e.g. genetic mosaicism, genomic imprinting), which might result in deviations from the classic, vertical transmission of Y chromosome haplotype from father to son [16].

### Case: reproductive cancers

The anomalies which result in reproductive dysfunction often predispose men to the development of other urologic ailments, including prostate cancer [17]. Indeed, just as genomic instability and Y chromosome deletions contribute to infertility, both can also contribute to carcinogenesis [13, 18]. Y chromosome deletions are one of the most common genetic anomalies among prostate cancer patients [19] and include the loss of various genes, including TSPY, PRY, EIF1AY, TMSB4Y, ZFY, BPY1, KDM5D, RBMY1A1, BPY2, and SRY [20–22]. Furthermore, there is a positive association between the number of gene losses and the stage/grade of prostate cancer [22].

TSPY is perhaps the most prominent Y-chromosome gene whose loss contributes to prostate cancer. Low copy numbers are associated with higher risk of prostate cancer [23, 24]. However, upregulation of products in the protocadherin gene family are involved in aggressive, apoptosis-resistant prostate cancers, as evidenced by cell lines which, after selection for apopotosis resistance, produce protocadherin-PC [25]. Furthermore, the protocadherin gene PCDH11Y, appears to be associated with aggressive prostate cancers [26, 27] but the haplogroup link is still under research. There is apparently little research regarding the effect of Y-haplotype on these prostate cancer-contributing factors (but see [28]). However, using Y-linked short tandem repeats (STRs), a study of Malaysian men suggests that prostate cancer development is more common among the haplotype CAAA [29]. Furthermore, a study among men of European descent suggests that the haplogroup E1b1b1c, which is more common among Ashkenazi Jews than other Europeans, might be associated with the development of prostate cancer [30]. Interestingly, PCDH11Y is the only gene under positive selection out of the 506 Ensembl genes in YDHS and it might have a deeper role on Y haplogroup and prostate cancer related questions.

In YDHS there was only one SNP linked to haplogroup E1b1b1c, namely V6. The gene involved in this mutation is ENSG00000092377, also known as TBL1Y by the HUGO gene nomenclature committee, translating into 4 proteins (3 splice variants). The OMIM entry linked to this gene is 400,033. TBL1Y is an X-degenerate gene that is a homolog of TBL1X [23]. The anatomical system for this gene is germinal center and the cell type is B-lymphocyte according to YDHS. There are altogether 19 variations for this gene out of which 3 are deleterious, 7 are benign and the rest do not have any prediction regarding the outcome. Interestingly NCBIs Variation Reporter shows only 3 variations. The gene TBL1Y is not under positive selection.

Gonadoblastoma, a germ cell tumor, is also Y-linked. Gonadoblastomas have a high frequency in XY females [31–33], suggesting a link between Y chromosome dysfunction and tumor development although it could be related to erroneous gender differentiation during the fetal period. Unlike prostate cancer, in which low TSPY expression is associated with high risk, high TSPY expression is a putative cause of gonadoblastoma [34, 35]. TSPY is also expressed in seminomas, a testicular germ cell tumor [34].

### Case: heart disease

Heart disease has a higher prevalence among males than females [36]. Although both male and female heart disease patients have altered gene expression from healthy patients, Y chromosome transcripts are dramatically upregulated among male Idiopathic Dilated Cardiomyopathy (IDCM) patients [37]. Of these genes, USP9Y is overexpressed in heart disease patients; among black populations in the UK, the most common USP9Y haplotype (TBL1YA USP9YA; 79.1 % prevalence) is protective against heart disease [38]. Although this genotype was not associated with reduced likelihood of heart disease where it occurred among white British men, this apparent lack of protection may be due to low statistical power owing to its infrequent occurrence among whites (4.9 % prevalence). Other haplotypes also affect heart disease susceptibility among British men of European origin; haplogroup I is associated with a 50 % higher risk of developing coronary artery disease than other lineages [39]. Haplogroup I is a common haplogroup in the UK, second only to R1b1b2 (prevalence 14.5-17.0 %, 70.0-72.7 %, respectively; [39]).

Interestingly, the predisposition of haplogroup I to coronary artery disease seems attributable to high levels of inflammatory immune responses rather than traditional coronary risk factors (e.g. lipid profiles; [39]). However, in a separate study, no significant association between haplotype and cardiovascular risk factor-associated SNPs were found among British men of European origin [40]. The haplogroup I contains 53 mutations in YDHS database and 14 of those are located inside genes giving rise to 69 proteins. The location of haplogroup I genes seems to be in the long arm q11.2 and its sub-cytobands. The common OrphaNet denominator for a vast majority of haplogroup I genes appears to be “partial chromosome Y deletion” (ID:1646). The haplogroup R1b1b2 was refactored to R1b1a2 by the ISOGG consortium in 2011. There are 10 mutations stored in YDHS database for R1b1a2 haplogroup and interestingly only one mutation hits a gene region, namely eukaryotic translation initiation factor 1A (EIF1AY). The small amount of repeats is surprising, only two (L1M4 and AluY) in the gene; one would expect a lot more based on the repeat rich nature of the chromosome Y. EIF1AY is not under positive selection and it codes for two proteins that have 3 unique variations altogether. The variation effect predicting algorithms were unable to determine whether these mutations were deleterious or not. SIFT predicted one tolerated and one deleterious mutation, whereas PolyPhen flagged two mutations unknown. Both of the programs failed to produce a prediction for the known reference SNP, rs58802313.

### Case: autism

Autism spectrum disorders are far more prevalent among males than females, ranging from 4:1 universally to 23:1 among the neurologically normal [41]. Thus, a role of Y-linked genes has long been suspected for autism and related disorders (reviewed by [42]). Indeed, there is a high prevalence of unusual SNPs on the Y-chromosome genes TBL1Y (transducin (beta)-like 1) and NLGN4Y (neuroligin 4, Y-linked) in autistic males [43]. Y-chromosome aneuploidy is associated with a higher than average rate of autistic disorders [44], with 28.3 % of XXYY males exhibiting autism [45]. There are 568 mutations in TBL1Y stored in YDHS database but it seems that haplogroup T has the most mutations (44) for TBL1Y gene. Haplogroup T is common in some parts of Asia, Southern Europe and in Africa. Interestingly haplogroup A, which is prevalent also in Africa had a very high amount of mutations (43) too, although it has a high number of mutations in gene regions (Fig. 2).

Results from studies examining the heritability of autism and its linkage to specific haplotypes are mixed, however. Among European-American males, 4 of 9 unnamed haplotypes were significantly over- or under-represented in normal vs. autistic groups [43], suggesting that certain haplotypes are protective or contributive, respectively. Anyhow, there was no reported relationship of haplotype with autism among French, Swedish, and Norse men [46]. The haplogroup I is the most common one for Nordic countries and northernmost Europe [47].

### Y-SNP distribution and diseases using YDHS database

**Fig. 4:**
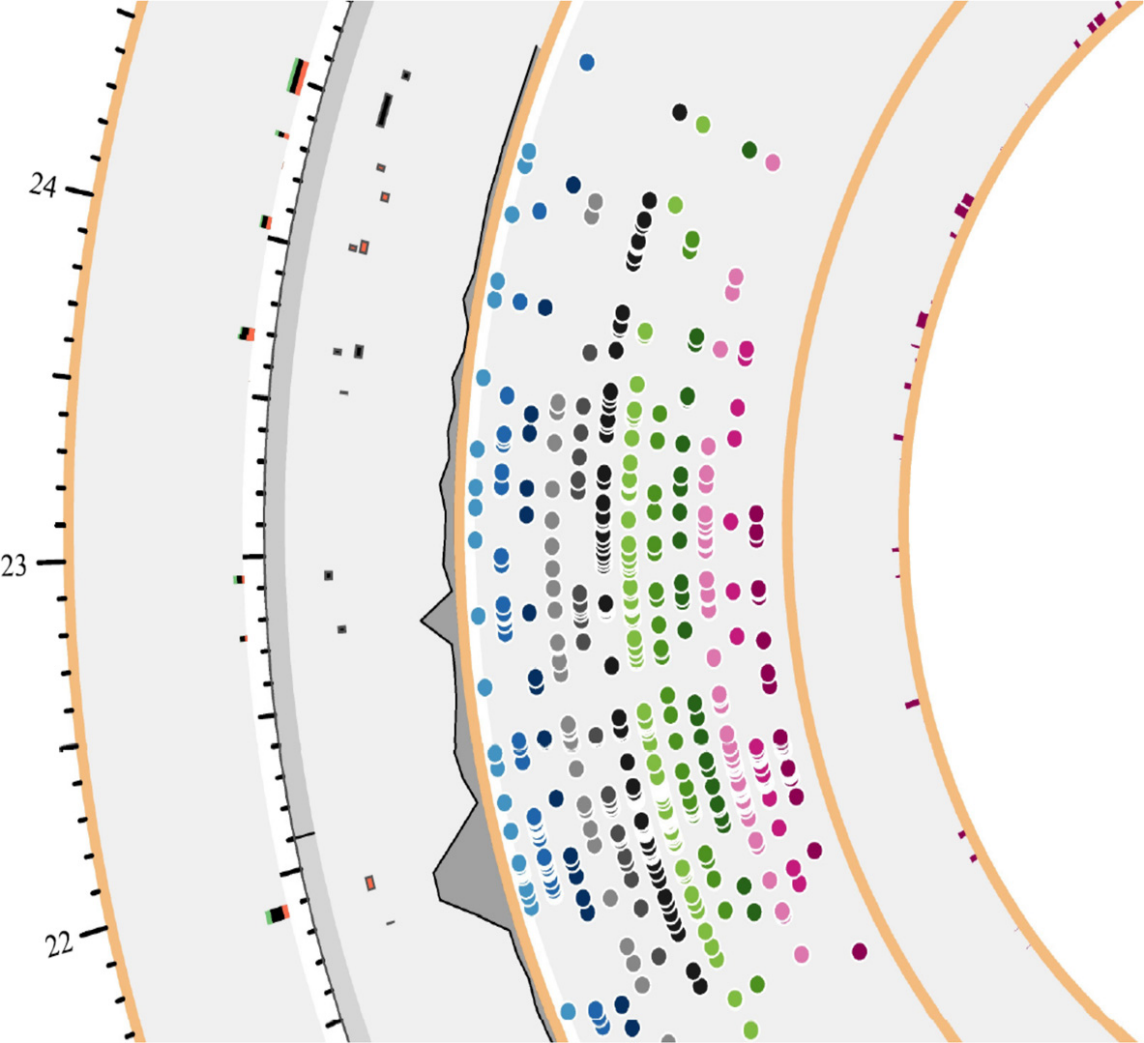
A more thorough look into Y-chromosome variant composition for the region between 22 Mb and 24 Mb

Visual inspection shows three distinct clusters of Y-SNP rich regions in Fig. 1 and since the location is shown in intervals of 1 Mb it is easy to identify the highly polymorphic regions. In this section we focus on area between roughly 22 and 24 Mb as visualized in Fig. 4 to illustrate the amount of information in YDHS. As explained in the Fig. 1 legend, the outer rim is reserved for the functional annotation part of the genes influenced by Y-SNP illustrated by bar diagram. Functional annotation is conducted using Gene Ontologies and the results are further split in to three color categories: biological process is viewed in red, molecular function in black and cellular component in green. The height of the bar and the abundance of these colors tells the user how much information the gene has e.g. higher bar means more ontologies are linked to it telling the annotation is more thorough. The color of the bar expresses the depth of the annotation process i.e. entirely green bar reveals the user that the vast majority of annotations are location thus cellular component related. This is categorization is advantageous since it offers an instant way to simplify complex datasets.

The layer below the annotation part illustrates the genes and the frequency of Y-SNPs as an area digram. The genes are color coded depending on the strand; genes located on positive strand are black and the ones on negative strand are red. The more there are Y-SNPs clustered, the higher the gray area of the plot, visualizing the highly polymorphic areas. The layer below the gene track is devoted to the Y-SNPs involved having the color schema explained in Fig. 1 legend.

The innermost layer is reserved for evidence of diseases linked to OrphaNet and/or OMIM. The higher the red bars are the more diseases are connected with the illustrated region. In Fig. 4 we have an interesting cluster of Y-SNPs in region 21,8-21,9 Mb. The genetic disorder linked to this region is OMIM #426000 and it could play a part in Y-linked spermatogenic failure as already explained in the AZF gene section. The gene involved is KDM5D. The KDM5D protein is, however, quite abundant according to PaxDB database (http://pax-db.org/#!protein/Q9BY66) which could also explain the high number of clustered mutations but it does not explain the Y chromosome link as the YDHS database does. Additional information can be found from Table 1 as the number of Gene Ontologies (55).

Currently there are 861 mutations stored in YDHS for KDM5D gene, and in terms of haplogroups the mutations are quite evenly distributed among haplogroups; mutation numbers per haplogroup vary between 7 to 49. The most common haplogroups were R1a and R1b with 49 and 35 mutations, respectively. The haplogroups are common in Europe, South and Central Asia and parts of Africa. R1a and R1b are not among the most mutation-rich haplogroups as can be seen in Fig. 3. Interestingly subclades deriving from R1a are very well represented in the Y-SNP pool e.g. R1a1a1b1a3b. The same order applies to Fig. 2, which provides Y-SNP distribution to gene areas solely, since our example is a functional gene.

## Conclusions

During its journey chromosome Y has had its ups and downs – from being called a boring “dud” [48] or genomic wasteland to the recent years’ rollercoaster ride of whether it is going to vanish from the face of the earth or restructure itself [49]. However despite the interest and efforts there has been very little research in embedding haplogroup information and variations to the genomic data regarding various diseases and conditions. At the time of writing this article there was no service or database for integrating Y chromosome haplogroups and genomic data from public resources. Integrating this data without a dedicated database would have required substantial manual work and bioinformatics expertise.

Now in YDHS, we have combined multiple tools under one resource that enables the user to e.g. browse through haplogroups and focus on certain SNPs distinct to that haplogroup and reveal where they are located and whether they have a link to a disease or condition and view the related genes and interactions. In addition, the user can run a set of predictor programs to see if the SNP is pathogenic or not and narrow down the geographic location of the haplogroup. We also provide text search to find articles where the SNP of interest was first introduced. The YDHS database provides an easy and convenient one-stop-shop to do translational research with chromosome Y without the need to browse around multiple databases or tackle with different data formats. As shown with the cases described here, haplogroups may provide an interesting insight to a number of genetic conditions.

## References

[1] Krausz C, Quintana-Murci L, Forti G (2004). Y chromosome polymorphisms in medicine. Ann Med.

[2] Ma K, Sharkey A, Kirsch S, Vogt P, Keil R, Hargreave TB (1992). Towards the molecular localization of the AZF locus - mapping of microdeletions in azoospermic men with 14 subintervals of interval-6 of the human Y-chromosome. Hum Mol Genet.

[3] Vogt PH, Edelmann A, Kirsch S, Henegariu O, Hirschmann P, Kiesewetter F (1996). Human Y chromosome azoospermia factors (AZF) mapped to different subregions in Yq11. Hum Mol Genet.

[4] Kunej T, Zorn B, Peterlin B (2003). Y chromosome microdeletions in infertile men with cryptorchidism. Fertil Steril.

[5] Plaseska-Karanfilska D, Noveski P, Plaseski T, Maleva I, Madjunkova S, Moneva Z (2012). Genetic causes of male infertility. Balk J Med Genet.

[6] McElreavey K, Quintana-Murci L (2003). Y chromosome haplogroups: A correlation with testicular dysgenesis syndrome?. Apmis.

[7] Rosser ZH, Zerjal T, Hurles ME, Adojaan M, Alavantic D, Amorim A (2000). Y-chromosomal diversity in Europe is clinal and influenced primarily by geography, rather than by language. Am J Hum Genet.

[8] Choi J, Koh E, Matsui F, Sugimoto K, Suzuki H, Maeda Y (2008). Study of azoospermia factor-a deletion caused by homologous recombination between the human endogenous retroviral elements and population-specific alleles in Japanese infertile males. Fertil Steril.

[9] Repping S, Skaletsky H, Brown L, van Daalen SKM, Korver CM, Pyntikova T (2003). Polymorphism for a 1.6-Mb deletion of the human Y chromosome persists through balance between recurrent mutation and haploid selection. Nat Genet.

[10] Underhill PA, Passarino G, Lin AA, Shen P, Lahr MM, Foley RA (2001). The phylogeography of Y chromosome binary haplotypes and the origins of modern human populations. Ann Hum Genet.

[11] Sato Y, Shinka T, Nakahori Y. Y chromosome haplogroup d2* lineage is associated with azoospermia in Japanese males. 2013(88):107. 10.1095/biolreprod.112.105718 23467741

[12] Paracchini S, Stuppia L, Gatta V, Palka G, Moro E, Foresta C (2000). Y-chromosomal DNA haplotypes in infertile European males carrying Y-microdeletions. J Endocrinol Invest.

[13] Aston KI, Carrell DT (2012). Emerging evidence for the role of genomic instability in male factor infertility. Syst Biol Reprod Med.

[14] Gazvani MR, Wilson EDA, Richmond DH, Howard PJ, Kingsland CR, Lewis-Jones DI (2000). Evaluation of the role of mitotic instability in karyotypically normal men with oligozoospermia. Fertil Steril.

[15] Wallerand H, Chabannes E, Bittard H (2001). Idiopathic male infertility and androgen receptors. Prog Urol.

[16] Jarvi K, Chitayat D (2008). The genetics you never knew: A genetics primer. Urol Clin North Am.

[17] Walsh TJ (2011). Male reproductive health and prostate cancer risk. Curr Opin Urol.

[18] Jangravi Z, Alikhani M, Arefnezhad B, Tabar MS, Taleahmad S, Karamzadeh R (2013). A Fresh Look at the Male-specific Region of the Human Y Chromosome. J Proteome Res.

[19] Sandberg AA (1992). Chromosomal abnormalities and related events in prostate cancer. Hum Pathol.

[20] Dasari VK, Goharderakhshan RZ, Perinchery G, Li L-C, Tanaka Y, Alonzo J (2001). Expression analysis of Y chromosome genes in human prostate cancer. J Urol.

[21] Lau Y-FC, Zhang J (2000). Expression analysis of thirty one Y chromosome genes in human prostate cancer. Mol Carcinog.

[22] Vergnaud G, Page DC, Simmler MC, Brown L, Rouyer F, Noel B (1986). A deletion map of the human Y-chromosome based on DNA hybridization. Am J Hum Genet.

[23] Skaletsky H, Kuroda-Kawaguchi T, Minx PJ, Cordum HS, Hillier L, Brown LG (2003). The male-specific region of the human Y chromosome is a mosaic of discrete sequence classes. Nature.

[24] Vijayakumar S, Hall DC, Reveles XT, Troyer DA, Thompson IM, Garcia D (2006). Detection of Recurrent Copy Number Loss at Yp11.2 Involving TSPY Gene Cluster in Prostate Cancer Using Array-Based Comparative Genomic Hybridization. Cancer Res.

[25] Chen MW, Vacherot F, de la Taille A, Gil-Diez-de-Medina S, Shen RQ, Friedman RA (2002). The emergence of protocadherin-PC expression during the acquisition of apoptosis-resistance by prostate cancer cells. Oncogene.

[26] Blanco P, Shlumukova M, Sargent CA, Jobling MA, Affara N, Hurles ME (2000). Divergent outcomes of intrachromosomal recombination on the human Y chromosome: male infertility and recurrent polymorphism. J Med Genet.

[27] Terry S, Queires L, Gil-Diez-de-Medina S, Chen M-W, de la Taille A, Allory Y (2006). Protocadherin-PC promotes androgen-independent prostate cancer cell growth. Prostate.

[28] Lindstrom S, Adami HO, Adolfsson J, Wiklund F (2008). Y Chromosome Haplotypes and Prostate Cancer in Sweden. Clin Cancer Res.

[29] Nargesi MM, Ismail P, Razack AHA, Pasalar P, Nazemi A, Oshkoor SA (2011). Linkage between Prostate Cancer Occurrence and Y-Chromosomal DYS Loci in Malaysian Subjects. Asian Pac J Cancer Prev.

[30] Wang ZM, Parikh H, Jia JP, Myers T, Yeager M, Jacobs KB (2012). Y chromosome haplogroups and prostate cancer in populations of European and Ashkenazi Jewish ancestry. Hum Genet.

[31] Hildenbrand R, Schröder W, Brude E, Schepler A, König R, Stutte HJ (1999). Detection of TSPY protein in a unilateral microscopic gonadoblastoma of a Turner mosaic patient with a Y-derived marker chromosome. J Pathol.

[32] Kersemaekers A-MF, Honecker F, Stoop H, Cools M, Molier M, Wolffenbuttel K (2005). Identification of germ cells at risk for neoplastic transformation in gonadoblastoma: An immunohistochemical study for OCT3/4 and TSPY. Hum Pathol.

[33] Page DC (1987). Hypothesis - a Y-chromosomal gene causes gonadoblastoma in dysgenetic gonads. Development.

[34] Lau Y-FC (1999). Gonadoblastoma, Testicular and Prostate Cancers, and the TSPY Gene. Am J Hum Genet.

[35] Salo P, Kääriäinen H, Petrovic V, Peltomäki P, Page DC, de la Chapelle A (1995). Molecular mapping of the putative gonadoblastoma locus on the Y chromosome. Genes Chromosomes Cancer.

[36] Schocken DD, Arrieta MI, Leaverton PE, Ross EA (1992). Prevalence and mortality rate of congestive heart failure in the United States. J Am Coll Cardiol.

[37] Heidecker B, Lamirault G, Kasper EK, Wittstein IS, Champion HC, Breton E (2010). The gene expression profile of patients with new-onset heart failure reveals important gender-specific differences. Eur Heart J.

[38] Russo P, Siani A, Miller MA, Karanam S, Esposito T, Gianfrancesco F (2008). Genetic variants of Y chromosome are associated with a protective lipid profile in black men. Arterioscler Thromb Vasc Biol.

[39] Charchar FJ, Bloomer LDS, Barnes TA, Cowley MJ, Nelson CP, Wang YZ (2012). Inheritance of coronary artery disease in men: an analysis of the role of the Y chromosome. Lancet.

[40] Chen XH, Rodriguez S, Hawe E, Talmud PJ, Miller GJ, Underhill P (2004). Evidence of admixture from haplotyping in an epidemiological study of UK Caucasian males: Implications for association analyses. Hum Hered.

[41] Miles JH, Hillman RE (2000). Value of a clinical morphology examination in autism. Am J Med Genet.

[42] Baron-Cohen S, Lombardo MV, Auyeung B, Ashwin E, Chakrabarti B, Knickmeyer R. Why Are Autism Spectrum Conditions More Prevalent in Males? Plos Biol [Internet]. 2011 Jun;9. Available from: ://WOS:000292191200008 10.1371/journal.pbio.1001081 PMC3114757 21695109

[43] Serajee FJ, Mahbubul Huq AHM (2009). Association of Y Chromosome Haplotypes With Autism. J Child Neurol.

[44] Mariner R, Jackson AW, Levitas A, Hagerman RJ, Braden M, McBogg PM (1986). Autism, mental retardation, and chromosomal abnormalities. J Autism Dev Disord.

[45] Tartaglia N, Davis S, Hench A, Nitnishakavi S, Beauregard R, Reynolds A (2008). A new look at XXYY syndrome: Medical and psychological features. Am J Med Genet A.

[46] Jamain S, Quach H, Quintana-Murci L, Betancur C, Philippe A, Gillberg C (2002). Y chromosome haplogroups in autistic subjects. Mol Psychiatry.

[47] Lappalainen T, Laitinen V, Salmela E, Andersen P, Huoponen K, Savontaus ML (2008). Migration waves to the Baltic Sea region. Ann Hum Genet.

[48] McKusick VA (1962). On the X chromosome of man. Q Rev Biol.

[49] Hughes JF, Skaletsky H, Brown LG, Pyntikova T, Graves T, Fulton RS, Dugan S, Ding Y, Buhay CJ, Kremitzki C, Wang Q, Shen H, Holder M, Villasana D, Nazareth LV, Cree A, Courtney L, Veizer J, Kotkiewicz H, Cho TJ, Koutseva N, Rozen S, Muzny DM, Warren WC, Gibbs RA, Wilson RK, Page DC. Strict evolutionary conservation followed rapid gene loss on human and rhesus Y chromosomes. Nature 483(7387):82–6 10.1038/nature10843 PMC3292678 22367542

